# Synchronization patterns reveal neuronal coding of working memory content

**DOI:** 10.1016/j.celrep.2021.109566

**Published:** 2021-08-24

**Authors:** Fahimeh Mamashli, Sheraz Khan, Matti Hämäläinen, Mainak Jas, Tommi Raij, Steven M. Stufflebeam, Aapo Nummenmaa, Jyrki Ahveninen

**Affiliations:** 1 Athinoula A. Martinos Center for Biomedical Imaging, Department of Radiology, Massachusetts General Hospital, Bldg. 149 13^th^ Street, Charlestown, MA 02129, USA; 2 Department of Radiology, Harvard Medical School, 25 Shattuck Street, Boston, MA 02115, USA; 3 Departments of Physical Medicine and Rehabilitation and Neurobiology, Northwestern University, 710 North Lake Shore Drive, Chicago, IL 60611, USA; 4 Lead contact

## Abstract

Neuronal oscillations are suggested to play an important role in auditory working memory (WM), but their contribution to content-specific representations has remained unclear. Here, we measure magnetoencephalography during a retro-cueing task with parametric ripple-sound stimuli, which are spectrotemporally similar to speech but resist non-auditory memory strategies. Using machine learning analyses, with rigorous between-subject cross-validation and non-parametric permutation testing, we show that memorized sound content is strongly represented in phase-synchronization patterns between subregions of auditory and frontoparietal cortices. These phase-synchronization patterns predict the memorized sound content steadily across the studied maintenance period. In addition to connectivity-based representations, there are indices of more local, “activity silent” representations in auditory cortices, where the decoding accuracy of WM content significantly increases after task-irrelevant “impulse stimuli.” Our results demonstrate that synchronization patterns across auditory sensory and association areas orchestrate neuronal coding of auditory WM content. This connectivity-based coding scheme could also extend beyond the auditory domain.

## In brief

Mamashli et al. use machine learning analyses of human magnetoencephalography (MEG) recordings to study “working memory,” maintenance of information in mind over brief periods of time. Their results show that the human brain maintains working memory content in transient functional connectivity patterns across sensory and association areas.

## Graphical abstract

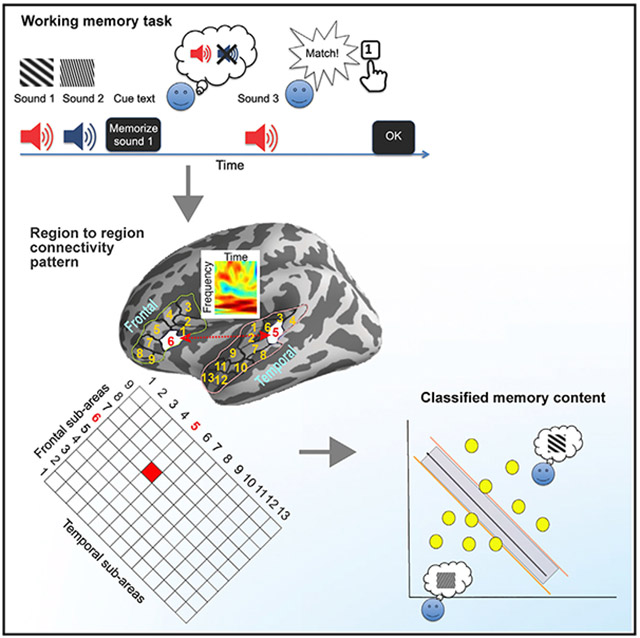

## INTRODUCTION

Auditory working memory (WM) refers to our capability to maintain and manipulate sound information in our minds over brief periods of time, which has co-evolved with the auditory-vocal communication skills that set humans apart in the animal kingdom (Aboitiz, 2018). How neurons maintain information in WM and how different parts of the brain contribute to this process continue to be unresolved and debated questions, irrespective of stimulus modality (Constantinidis et al., 2018; Stokes, 2015; Xu, 2017). A specific complication in the auditory domain has been that many of the available WM tasks have been based on complex stimuli that allow or even facilitate non-auditory WM maintenance strategies (Kaiser et al., 2003, 2009; Lutzenberger et al., 2002), such as verbal rehearsal (Smith and Jonides, 1997). The fundamental mechanisms of how purely auditory attributes are maintained thus constitute one of the least understood aspects of human WM (Scott and Mishkin, 2016).

WM research has long been influenced by a hypothesis that there is a dedicated area of prefrontal (PFC) or posterior parietal (PPC) cortices, which maintains information via the sustained firing of neurons (Fuster and Alexander, 1971; Goldman-Rakic, 2011; Smith and Jonides, 1999; Xu, 2017). However, it is becoming increasingly clear that activation patterns that carry the information of WM content occur at many areas of the brain (Christophel et al., 2017), ranging from early auditory or visual cortices to the highest cognitive areas (Bigelow et al., 2014; Christophel et al., 2012; Gottlieb et al., 1989; Grimault et al., 2009; Huang et al., 2016; Kumar et al., 2016; Linke et al., 2011; Ng et al., 2014; Scott et al., 2014; Serences, 2016; Sreenivasan et al., 2014; Uluç et al., 2018; Wolff et al., 2020). Many current theories thus view WM as an emergent property of functionally interconnected brain areas that represent different sensory, perceptual, and cognitive stages of the task-relevant content (Christophel et al., 2017; Postle, 2006). However, although these distributed models are gaining wide support, relatively few studies have examined information content in interregional patterns of brain activity per se during WM maintenance (Salazar et al., 2012; Soreq et al., 2019).

An ideal way to examine the role of interregional functional connectivity patterns in human WM is to analyze interregional phase synchronization of neuronal oscillations estimated from magnetoencephalography (MEG) signals. In previous visual studies, indices of long-range synchronization effects that depend on task attributes such as the amount of maintained information, or “WM load,” have been reported both using human MEG (Daume et al., 2017; Palva et al., 2010; Sato et al., 2018) and non-human primate neurophysiology (Buschman et al., 2012; Salazar et al., 2012). At the same time, accumulating neurophysiological evidence suggests that functional collectivity of more local neuronal networks, mediated by bursts of neuronal oscillations that modulate and refresh synaptic plasticity, play a crucial role in supporting WM maintenance (Lundqvist et al., 2016; Miller et al., 2018). However, whether phase synchronization across the broader network of brain areas involved in human WM carries information of its memory content is still uncertain.

Here, we tested the hypothesis that auditory WM content is encoded and maintained in transient changes of functional connectivity between brain areas involved in auditory perception and cognition. To this end, we examined frequency-specific changes in phase synchronization of neuronal oscillations using MEG, a non-invasive measure of synaptic currents in the human brain. Unlike EEG, MEG readily dissociates signals from the auditory cortices and frontocentral regions in the sensor space (Hamalainen et al., 1993), which significantly facilitates the cortical source estimation needed for our hypothesis testing. To ensure that the effects reflect purely auditory WM, we designed a task with dynamic ripple sound stimuli, which are spectrotemporally similar to human vocalizations but resist non-auditory memory strategies (Visscher et al., 2007). We developed a multivariate machine-learning pipeline to predict the ripple-sound content maintained in WM using the patterns of subregional functional connectivity as well as spectral and temporal brain activity measures. In addition, we tested for the existence of “activity silent” WM representations in auditory cortices (Mongillo et al., 2008; Stokes, 2015) by examining the content specificity of oscillatory power patterns elicited to task-irrelevant impulse stimuli (Rose et al., 2016; Wolff et al., 2015, 2017, 2020).

## RESULTS

Using machine learning, we show that phase synchronization patterns between subregions of auditory and frontoparietal cortices predict the content of auditory WM with high accuracy. In most of these functional connectivity patterns, the decoding accuracy of memory content remained stable across the studied maintenance period. Furthermore, the connections that revealed the WM content during the later stage of maintenance consisted of a subset of those found during the earlier part of maintenance. In addition to these connectivity-based results, we found indices of activity silent WM representations in auditory cortices, as suggested by enhanced decoding of memorized sound content from patterns of oscillatory power after task-irrelevant impulse sounds.

### Behavioral performance

**Figure 1.**
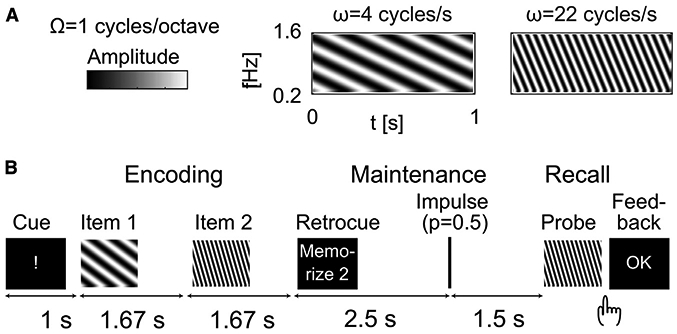
Auditory stimuli and WM tasks (A) Time-frequency representations of 2 moving ripple sounds, modulated across time (ripple velocity, ω cycles/s) and frequency (Ω cycles/octave). (B) Trial design. After an alerting cue, subjects heard 2 ripple sound stimuli (i.e., memory items) in a row. A brief visual cue then followed, to instruct which of the previous items was to be actively memorized for a period of 4 s. After hearing the probe, the subject was asked to press one button (“yes”) if the probe matched the relevant item and another(“no”) if it did not. In half of the trials, a brief broadband auditory noise burst called “impulse stimulus” was presented during the maintenance period, to help decode item-specific activations.

Auditory WM was examined using a “retro-cueing” paradigm, a strategy that helps control for the differing accounts of recent stimulus history (i.e., passive sensory memory) and actively maintained WM content (Kumar et al., 2016; Rose et al., 2016; Sprague et al., 2016; Uluç et al., 2018; Wolff et al., 2017) (Figure 1). The memory items consisted of six ripple velocities separated by 1.5 of their just noticeable differences (JNDs), which was determined in a separate session to control for individual differences in sound discrimination (Visscher et al., 2007). MEG data were measured from 20 participants while subjects were performing the WM task. All of the subjects were capable of performing the task according to the instruction. The mean proportion of correct responses was 0.84 (standard deviation = 0.1).

### Auditory WM content-specific functional connectivity patterns

**Figure 2.**
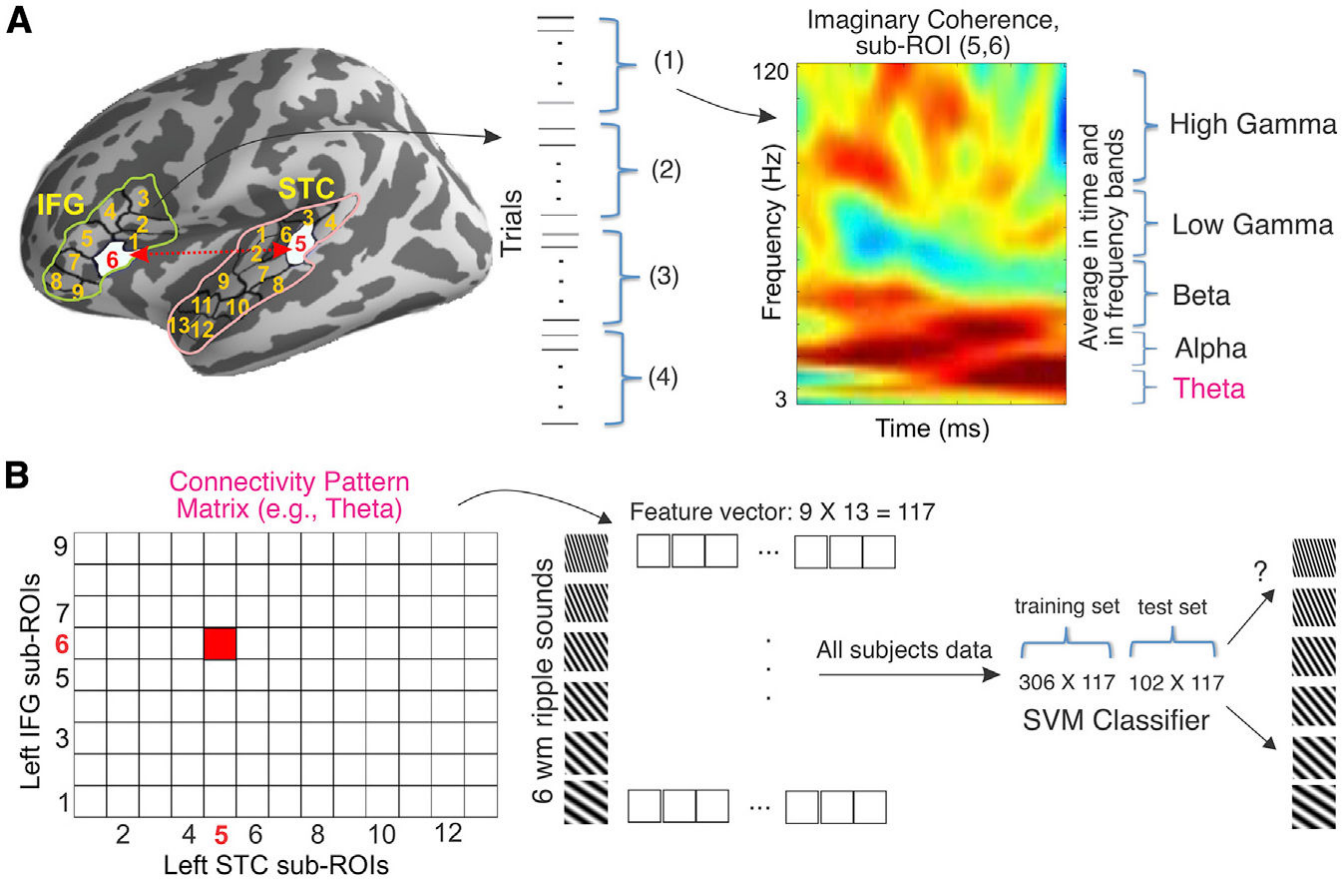
Schematic of multivariate pattern analysis (MVPA) using the connectivity pattern between 2 examples of ROIs We used an automatic routine to break each large ROI into smaller, approximately equal-size sub-ROIs (Mamashli et al., 2019a) to increase the spatial specificity, allowing us to capture the spatio-spectral WM activation variability. (A) Imaginary coherence (ImCoh) was estimated between 9 × 13 sub-ROIs pairs within STC and IFG across each ¼ of the total number of epochs. An example of the ImCoh is shown at right. The ImCoh were averaged in early (0.5–1.25 s) and late (1.25–2 s from memory cue) time windows and each frequency band. (B) The ImCoh value of each sub-ROI pair was used to generate the connectivity matrix. The connectivity pattern matrix was then converted to a vector (e.g., here consisting of 9 × 13 = 117 features) to classify the 6 sound classes. We then combined all subjects’ data, fed them into a linear support vector machine, trained the model on the data from 75% of the subjects and tested it on the remaining subjects. For cross-validation, we permuted the data 100 times and replicated the classification. The accuracy was used as the performance metric. The related confusion matrices are in Figures S2 and S3. For further details of the method, see STAR Methods.

We selected six regions of interest (ROIs) based on the previous studies on auditory WM (Buchsbaum et al., 2005; Crottaz-Herbette et al., 2004; Huang et al., 2013; Kumar et al., 2016; Rodriguez-Jimenez et al., 2009; Uluç et al., 2018; Vallar et al., 1997), as specifically defined by using the Freesurfer Desikan parcellation (Desikan et al., 2006). These ROIs included the superior temporal cortex (STC; superior temporal and Heschl’s gyri combined) and inferior frontal gyrus (IFG), as well as the caudal middle frontal (CMF), lateral orbitofrontal (LOF), rostral middle frontal (RMF), precentral (PC), and supramarginal (SM; overlapping with intraparietal lobule [IPL]) areas in each hemisphere. Given that different parts of STC are sensitive to different spectrotemporal properties (Schönwiesner and Zatorre, 2009), we hypothesized that the memorized ripple-sound content is represented in the subregional phase-synchronization patterns between the auditory cortex and other ROIs. Therefore, we focused on the connections between STC and other ROIs to tap into the role of networks involving sensory cortices in WM. We developed an approach that uses sub-ROI functional connectivity patterns to predict the WM content with a linear support vector machine (SVM) classifier (Figure 2). As the functional connectivity measure, the imaginary part of the coherence (ImCoh) between sub-ROI pairs (Figure 2A) was used to minimize spurious connectivity due to the field spread effect (Nolte et al., 2004). We considered early (0.5–1.25 s from memory cue) and late (1.25–2 s) time windows in the maintenance period. The data from all of the subjects were merged together within the classification, which was cross-validated by permuting the data 100 times. This means training the model with data from 75% of the subjects and testing on the remaining data. The statistical significance of the classification accuracy was determined by comparing the original accuracy with a null distribution created by using a randomized classifier by permuting the labels 500 times. To control for multiple comparisons, from each permutation, the maximum statistical value across the studied 60 connections (12 STC-ROI pairs in 5 frequency bands) was assigned to this null distribution. To further test the validity of our results, we conducted a similar connectivity-based decoding analysis with the visual cortex area lateral occipital cortex (LOC) as the seed region (for further details of the statistical analyses, see STAR Methods).

**Figure 3.**
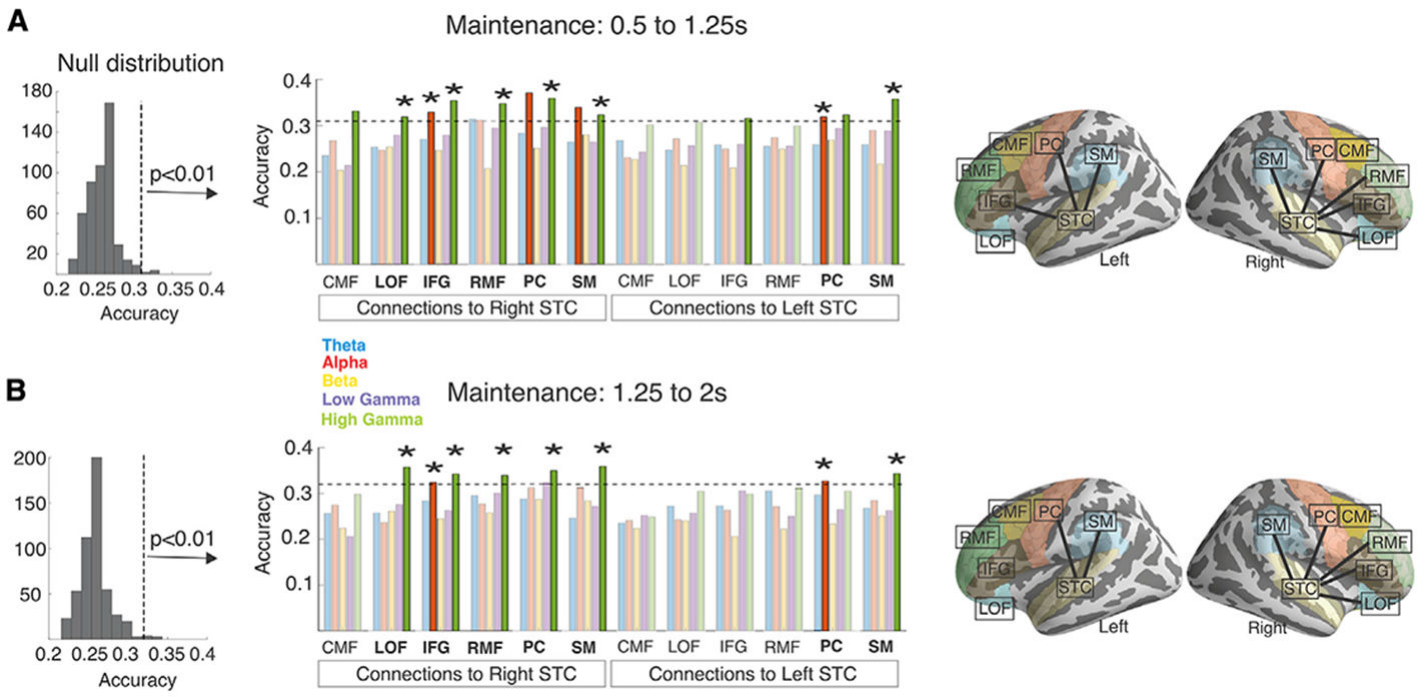
Evidence for connectivity-based auditory WM representations Decoding accuracy in the connectivity-based decoding analysis at the 5 studied frequency bands during (A) the earlier (0.5–1.25 s) and (B) the later (1.25–2 s) WM maintenance time windows. The left panel shows the null distributions of maximum statistics for each time window, created using classifiers with randomized stimulus-item labels. The thresholds of significance are marked with vertical dashed lines. The center panel shows the decoding accuracy values for each connection and frequency within each time window. Those above the threshold (horizontal dashed line) were deemed statistically significant. The connections with the most stable decoding accuracy, revealing the WM content both during the early (A) and later (B) time windows, are labeled with an asterisk. The rightmost panel shows the anatomical connections that showed significant decoding accuracy in at least 1 of the frequency bands. For further details of the analysis, see the subsections Method details and Quantification and statistical analysis in the STAR Methods.

**Figure 4.**
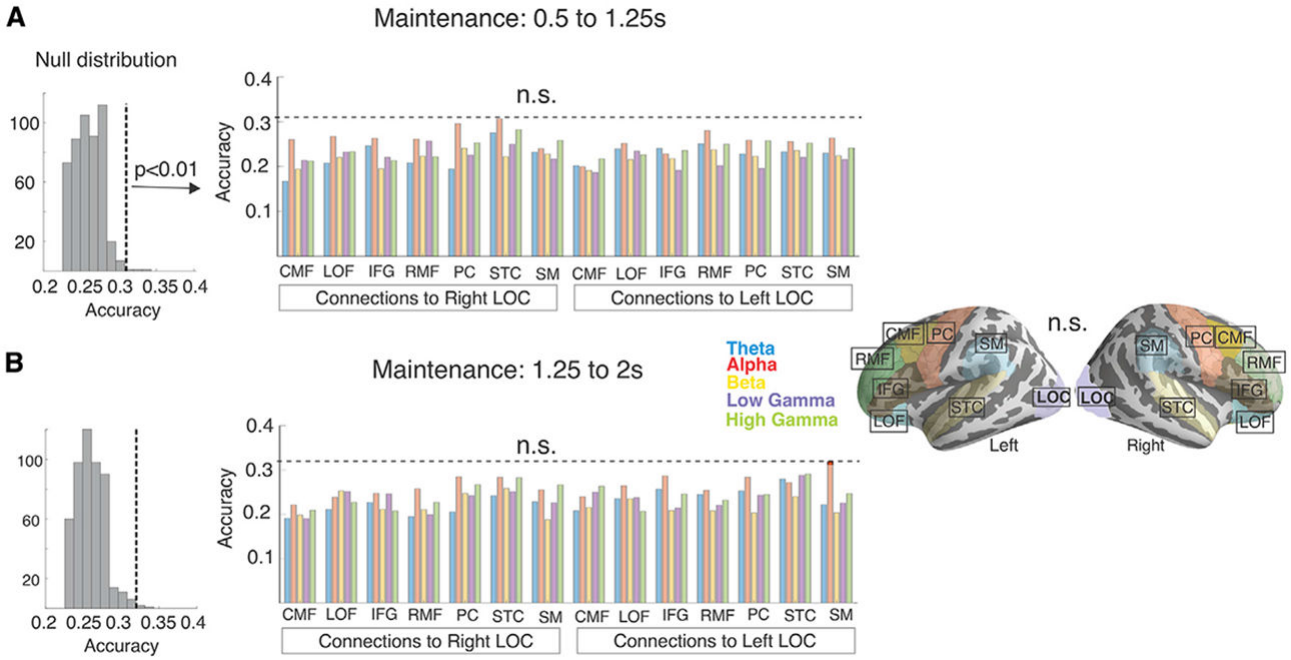
No significant effects were observed in a control analysis, which used connectivity patterns to the visual cortex area LOC for decoding the memorized sound content (A) Earlier time window (0.5–1.25 s) of WM maintenance. (B) Later time window (1.25–2 s) of WM maintenance. The left panel shows the null distributions of maximum statistics for each time window, created using a classifier with randomized sound-content labels. The dashed lines show the critical values (i.e., the threshold) of statistical significance for each time window. The center panels show the accuracy values for each connection and each frequency, none of which were statistically significant. The rightmost panel displays the location of the LOC seed and other ROIs. For further details of the analysis, see the subsections Method details and Quantification and statistical analysis in the STAR Methods.

During maintenance, decoding accuracies were significantly above chance level in 12 frequency-specific connections within the early time window (Figure 3A) and in 8 connections within the later time window (Figure 3B). Significant connectivity-based decoding results were found in both hemispheres at the alpha and gamma ranges, with the strongest effects emerging at the high gamma band in the right hemisphere (for details of the statistical inference, see STAR Methods). Importantly, all connections that revealed WM content within the late time window showed significant decoding results also within the early time window (labeled with an asterisk in Figure 3). In the right hemisphere, WM content was stably decodable from both time windows from the high gamma-band connection patterns between STC versus LOF, IFG, RMF, CMF, and PC, as well as from the alpha-band connection patterns between STC and IFG. In the left hemisphere, the WM content could be stably decoded from the alpha-band STC-PC and high gamma-band STC-SM connectivity patterns (Figure 3). In the control analysis with LOC as the seed, none of the estimated decoding accuracies was significantly above chance level (Figure 4; for details of the statistical inference, see STAR Methods). The results of additional connectivity-based analyses, which decoded the stimulus content during the WM encoding, are presented in Figure S1. Examples of confusion matrices related to the decoding analyses during the maintenance period are shown in Figures S2 and S3.

**Figure 5.**
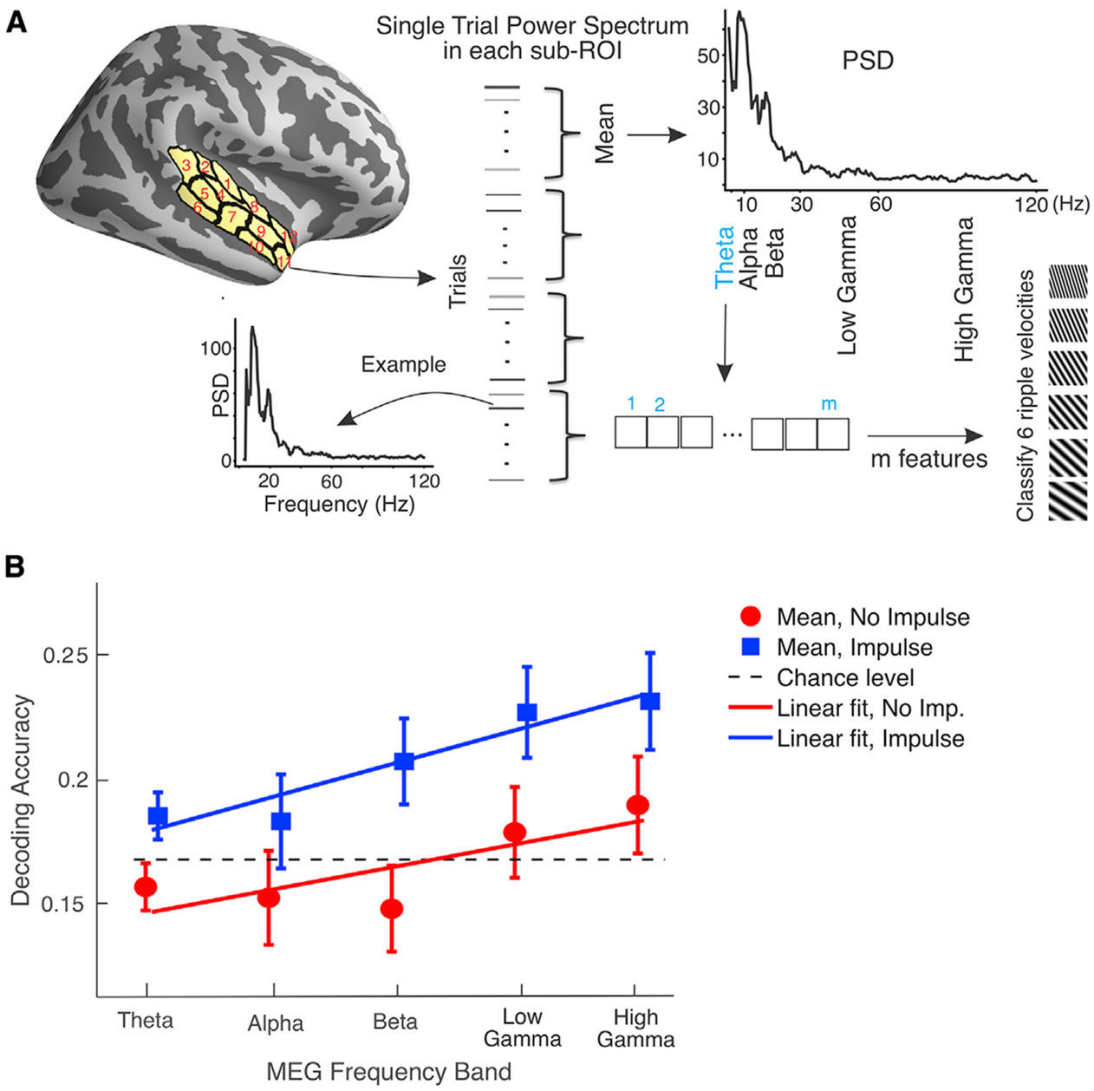
MVPA using oscillatory power and impulse sound effect (A) Schematic display of MVPA using STC oscillatory power to decode activity-silent WM representations in auditory areas. Single-epoch power was estimated within each sub-ROI and then averaged across ¼ of the epochs within frequency bin. At each separate frequency band, the bilateral STC power pattern included 25 sub-ROIs (i.e., 12 in right-STC and 13 in left-STC), which were entered into a band-specific SVM classifier with 4-fold cross-validation. (B) MVPA decoding accuracy at 5 frequency bands in theta, alpha, beta, low, and high gamma bands in response to impulse and no-impulse sound. According to our linear mixed-effects model, the decoding accuracy was significantly enhanced by the impulse sound. The error bars show the standard error of the mean. For further details of the analysis, see the subsections Method details and Quantification and statistical analysis in the STAR Methods.

To rule out power-related biases in our coherence estimates, we conducted another control analysis, in which the power values in each ROI were used as features in the SVM classifier (Figure 5A). As expected, in contrast to the connectivity-based analysis, the control analysis aiming to classify the memorized ripple-sound content based on oscillatory power patterns at the same five frequency ranges provided no significant results in any of the ROIs (for details of the statistical inference, see STAR Methods).

### Probing the activity-silent WM traces in auditory cortex areas

Recent electroencephalography (EEG) studies suggest that task-irrelevant impulse stimuli help amplify the readout of neural memory traces from distributed brain activation patterns (Rose et al., 2016; Wolff et al., 2017, 2020). In addition to the connectivity -analysis, we therefore probed for activity silent WM representations in the auditory cortex by examining content-related changes of responses to task-irrelevant auditory impulse stimuli, which were unrelated to the stored attributes, per se: a 50-ms white-noise burst was randomly presented in 50% of the trials after 2.5 s into the delay period to induce a brief transient activation in auditory cortices (Figure 1). We then attempted to decode which of the six ripple sound items was maintained in WM from the frequency band x sub-ROI patterns of oscillatory power in left and right STC, with versus without the impulse sound (Figure 5A). The effect of impulse sound was tested by using a linear mixed effect (LME) model with fixed effect factors “impulse sound” (with versus without) and the MEG frequency band center frequency, as well as the random effect of subject identity, using the MATLAB function fitlme (for details of the statistical inference, see STAR Methods). In support of our hypothesis, the content of auditory WM could be decoded significantly more accurately from STC from trials that included the impulse sound versus those not including the impulse sound (*t*_187_ = 4.0, *p_Bonferroni_* = 0.001; Figure 5B). The best-fitting model furthermore suggested that the decoding accuracy generally increased as a function of the increasing MEG frequency band (*t*_187_ = 3.1, *p*_*Bonferroni*_ = 0.03; Figure 5B). This suggests that the impulse sound improves the decoding accuracy of WM content in STC areas that encompass the human auditory cortex.

We hypothesized that the readout of WM content would be enhanced by the impulse sound specifically in auditory cortices. However, in a confirmatory control analysis, we tested the effect of the impulse sound also in the frontal and parietal ROIs using a sub-ROI power pattern of spectral power in the five frequency bands (Figure 5A). We corrected for multiple comparisons with Bonferroni correction across 13 t-statistics. In contrast to the significant effects bilateral STCs, the impulse sound did not improve the decoding accuracy in our parietal and frontal ROIs. The analysis procedure is described in detail in the STAR Methods.

### Power variation during maintenance

A recent study in auditory WM examined the sustained activity in different frequency bands and areas during the maintenance period using local field potential data (Kumar et al., 2021). We therefore investigated oscillatory power variation during maintenance in all ROIs. We found that the power during maintenance shows sustained activity in alpha, beta, and low gamma in early time periods, while at later time windows, this pattern is present in the high gamma band (Figures S4 and S5; for further details of the statistical inference, see STAR Methods).

### Temporal pattern decoding

The auditory WM content could be classified significantly above chance level also from the temporal pattern of the MEG source activity during WM encoding (Figure S6), but no significant effects were found during maintenance. The details of the statistical analyses are presented in the STAR Methods.

### Behavioral relevance of functional connectivity during WM maintenance

Our support vector regression (SVR) and permutation testing analyses suggest that each subject’s behavioral WM performance can be predicted based on high-frequency STC-frontoparietal synchronization patterns, which in our main analysis contained information of WM content (see Figure S7). The details of statistical inference are described in the STAR Methods.

### Similarity between WM encoding and maintenance

Following the synaptic model of WM, we hypothesized that maintaining an item in auditory WM involves the same functional network that is used during encoding. To test this hypothesis, we trained a linear SVM classifier using the connectivity features of WM encoding and tested it with the connectivity pattern during WM maintenance. None of the connections were significantly above the chance level that was determined using the permutation method.

## DISCUSSION

Our results provide evidence that parametric attributes of auditory WM content are represented in behaviorally relevant neuronal phase-synchronization patterns, which connect auditory areas of STC with inferior-lateral frontoparietal cortices. According to our machine-learning analyses, which used rigorous between-subject cross-validation and non-parametric permutation testing, most of these connectivity-based WM representations remained stable across the studied maintenance period. Stable and significant connectivity-based decoding results were found at the alpha and high gamma bands in both hemispheres, with the strongest WM content representations emerging at the high gamma band in the right hemisphere. These findings support a hypothesis that auditory WM content is maintained in long-range synchronization patterns of neuronal oscillations.

It has long been debated whether WM involves a dedicated storage region or whether the maintenance process is distributed across multiple brain areas and hierarchical levels (for reviews, see Christophel et al., 2017; Goldman-Rakic, 2011; Postle, 2006; Stokes, 2015; Xu, 2017). Our results provide an alternative perspective to this theoretical question: WM maintenance could be supported by a broader interregional connectivity architecture, in which each dynamic ripple sound memory is represented by a content-specific combination of functional connections across different aspects of auditory cortex, premotor cortex, and frontoparietal association areas. The content specificity of these connectivity patterns could be built upon an intrinsic connectivity topography in which the “best ripple velocity” differs across different subareas of the auditory cortex (Massoudi et al., 2015; Schönwiesner and Zatorre, 2009) and also across frontoparietal neurons that are connected to different parts of the auditory cortex (Fritz et al., 2010). This connectivity-based coding hypothesis receives indirect support from a recent fMRI study, according to which the content of human visual WM can be decoded more precisely from functional connectivity than local hemodynamic activation patterns (Soreq et al., 2019).

The theoretical idea that WM content could be represented in functional connectivity has been described previously at the level of a local network of nearby neuronal units (Barak and Tsodyks, 2014; Mongillo et al., 2008; Stokes, 2015). A candidate mechanism for such local connectivity-based effects is short-term synaptic plasticity (STSP): During encoding, the stimulus-driven activity temporarily changes the synaptic efficacy within the neural network, which leaves behind a temporary synaptic memory trace via STSP (Zucker and Regehr, 2002). These STSP effects could then change the functional connectivity of the neural network and result in a transient item-specific circuit for the WM memoranda (Erickson et al., 2010; Stokes, 2015). It is possible such effects could also modulate the intrinsic connectivity of larger-scale networks that are observable in MEG source estimates. In other words, the content specificity of large-scale functional connectivity, which we observed in the linear classifier analysis of MEG source estimates, could reflect an altered state of the underlying synaptic connections.

Content-specific interregional synchronization in WM could also be intuitively explained by the communication through coherence theory (Fries, 2005). According to this theory, frequency-specific coherence provides a mechanism for information transfer by phase-aligning periods of excitability to maximize the opportunity for communication (Fries, 2005). During WM encoding, the intrinsic oscillatory activity of groups of neurons, which are sensitive to the to-be-remembered auditory attributes, could become synchronized or “entrained.” During maintenance, the entrained (interregional) oscillatory activity could then increase the temporal coherence of activations that, for example, help periodically refresh synaptic traces of the maintained information (for a review, see Miller et al., 2018).

In auditory cortices, we found a significant increase in the decoding accuracy of the memory content after task-irrelevant impulse sounds, when compared to maintenance trials with no such impulse stimuli. This finding is broadly consistent with previous EEG studies of activity silent representations in visual (Rose et al., 2016; Ten Oever et al., 2020; Wolff et al., 2017, 2020) and auditory WM (Wolff et al., 2020). However, as the previous non-invasive human studies were focused on the EEG electrode space analysis, the underlying sources of activity silent WM representations, and particularly the contribution of sensory areas, had remained ambiguous. Our MRI-constrained cortical MEG source analysis results thus significantly extend these previous findings by showing that activity silent WM maintenance takes place in the sensory areas of the human cortex as well.

In our connectivity-based decoding analyses, we found content-specific WM effects at alpha and high gamma ranges. Previous studies in both visual (Howard et al., 2003; Medendorp et al., 2007; Palva et al., 2010; Roux et al., 2012; van Vugt et al., 2010) and auditory domains (Kaiser et al., 2008; Lutzenberger et al., 2002) have suggested functional specificity of different oscillatory frequency ranges. In these studies, gamma band oscillations have often been associated with active maintenance of WM information (Roux and Uhlhaas, 2014). Previous neurophysiological work suggests that gamma activity is closely linked to spiking patterns that carry information about WM memoranda (Lundqvist et al., 2016). Although often considered predominantly a local phenomenon (Fries et al., 2007), gamma-range synchronization patterns occur also across longer distances (Buzsáki and Schomburg, 2015), including between the primate PFC and sensory areas during active attention tasks (Gregoriou et al., 2009). In contrast, alpha-band activity has been linked to protecting WM items from non-relevant information (Roux and Uhlhaas, 2014). Our results suggest that these frequency-specific changes in functional connectivity show content-specific variability, which can be decoded using machine-learning techniques.

In conclusion, our results provide converging evidence that auditory WM information is maintained in patterns of functional connectivity between subregions of frontoparietal and temporal cortices. The content specificity of these connectivity-based WM representations fundamentally relies on sensory areas of the human auditory cortex. It is conceivable that the principles of our connectivity-based perspective on the neural coding of WM extend beyond the auditory domain.

## STAR★METHODS

### RESOURCE AVAILABILITY

#### Lead contact

Further information and requests for resources should be directed to and will be fulfilled by the Lead Contact, Jyrki Ahveninen, jahveninen@mgh.harvard.edu.

#### Materials availability

This study did not generate new materials.

#### Data and code availability

- Deidentified MEG ImCoh data utilized in our connectivity-based analyses to yield our main results are available at https://dataverse.harvard.edu/citation?persistentId=doi:10.7910/DVN/I307DS.
- Custom code utilized to generate our results is available at https://zenodo.org/record/5112421.
- Any additional information required to reanalyze the data reported in this paper is available from the Lead Contact upon request.

### EXPERIMENTAL MODEL AND SUBJECT DETAILS

#### Human participants

A total of 20 healthy adult participants with no self-reported hearing deficits and (corrected-to-) normal vision were included (age 22-39 years, 12 women). The subjects’ capability to detect and discriminate the auditory stimuli were confirmed in a brief behavioral assessment before the MEG session. All procedures were approved by the Institutional Review Board of Massachusetts General Hospital. All subjects gave their informed consent before participating in the study.

### METHOD DETAILS

#### Stimuli and WM task

The majority of previous studies on auditory WM use stimuli that allow non-auditory maintenance strategies. Here, to eliminate verbal and other non-auditory rehearsal strategies, we used moving ripple sounds, which are spectrotemporally similar to speech but not contaminated by semantic properties or perceptual categories (Visscher et al., 2007) (Figure 1A). An individualized set of 17 stimuli with different ripple velocities, separated by intervals of **Δ**ω = 0.5 × the just noticeable difference (JND) were generated for each subject in a separate behavioral session, to control for individual differences in sound discrimination (Visscher et al., 2007). The dynamic ripple sounds were generated by superimposing 20 sinusoids/octave ranging from *f*_0_ = 0.2 kHz to *f* = 1.6 kHz. Their intensity at any time and frequency was defined by *s(g,t*) = *D*_*0*_ + *D* cos[2π(*ωt* + Ω*g*)) + *ψ*], where g = log(*f/f_0_*), D is the modulation depth, and ψ is the phase of the ripple (duration = 1 s, Ω = 1 cycles/octave). JND of ω was approximated as the minimally detectable base 2 logarithmic ripple-velocity interval within a range of 3-48 cycles/s based on an adaptive 2 down/1 up staircase algorithm. The sounds were delivered at a comfortable level via a headphone.

A “retro-cueing” paradigm was utilized to control for the differing accounts of recent stimulus history and actively maintained WM content (Kumar et al., 2016; Rose et al., 2016; Sprague et al., 2016; Uluç et al., 2018; Wolff et al., 2017) (Figure 1B). The subject was first presented with two sound items in a row. A subsequent retro-cue indicated which of the two items was to be maintained in memory. Four seconds after the retro-cue, the subject heard a probe stimulus and was asked to press one button if the probe matched the relevant item and another button if not. In 50% of the trials, the probe matched the maintained item. In 50% of the remaining non-matching trials (25% of total count), the probe matched the irrelevant item, to confirm that subjects were maintaining only the relevant item. Notably, the subject was not informed that whereas the probes were selected from the entire individualized pool of 17 possible stimuli, the pairs of items consisted of only 6 possible classes. To increase the physical variability to minimize any long-term learning effects, there was also a half-JND offset between the possible relevant versus irrelevant item classes. Finally, in half of the trials, a task-irrelevant impulse stimulus, a 50-ms white-noise burst, was randomly presented 2.5 s into the delay period. This impulse stimulus was utilized to tap into the hidden states of WM, as inspired by previous EEG studies (Rose et al., 2016; Wolff et al., 2017, 2020).

#### Structural MRI data acquisition and processing

T1-weighted anatomical images were obtained for combining anatomical and functional data using a multi-echo MPRAGE pulse sequence (TR = 2530 ms; 4 echoes with TEs = 1.69, 3.55, 5.41, 7.27 ms; 176 sagittal slices with 1 × 1 × 1 mm^3^ voxels, 256 × 256 mm^2^ matrix; flip angle = 7°) in a 3T Siemens Prisma whole-body MRI scanner (Siemens Medical Systems, Erlangen, Germany) using a 64-channel head and neck coil. Cortical reconstruction and parcellations for each subject were generated using Freesurfer (Dale et al., 1999; Fischl et al., 1999). After correcting for topological defects, the cortical surfaces were triangulated with dense meshes with ~130 000 vertices in each hemisphere. For visualization, the surfaces were inflated, thereby exposing the sulci (Dale et al., 1999).

#### MEG data acquisition

MEG data were acquired inside a magnetically shielded room (IMEDCO AG, Haegendorf, Switzerland) using a whole-head Vector-View MEG system (MEGIN Oy, Helsinki, Finland), comprised of 306 sensors arranged in 102 triplets of two orthogonal planar gradiometers and one magnetometer. The signals were filtered between 0.1 and 200 Hz and sampled at 1000 Hz. The position and orientation of the head with respect to the MEG sensor array was recorded continuously with help of four head position indicator coils. To allow co-registration of the MEG and MRI data, the locations of three fiduciary points (nasion and pre-auricular points) that define a head-based coordinate system, a set of points from the head surface, and the sites of the four head position indicator coils were digitized using a Fastrak digitizer (Polhemus) integrated with the Vectorview system. MEG Data were recorded in four 24-minute runs, with 96 trials within each run. The ECG and electrooculography signals were recorded simultaneously to identify epochs containing heartbeats as well as vertical and horizontal eye movement and blink artifacts. During data acquisition, online averages were computed from artifact-free epochs to monitor data quality in real time. All offline analysis was based on the saved raw data. In addition, 5 minutes of data from the room void of a subject were recorded before each experimental session for noise estimation purposes.

#### MEG data preprocessing

MEG data were spatially filtered using the signal space separation method (SSS, Elekta-Neuromag Maxfilter software) to suppress noise generated by sources outside the brain (Taulu et al., 2004). The SSS method also corrects for head motion between and within runs (Taulu et al., 2004). Cardiac and ocular artifacts were removed by signal space projection (SSP) (Khan et al., 2018; Mamashli et al., 2019b). Data were visually browsed and sufficient number of SSP (between 1-4) were selected separately to remove cardiac and ocular artifacts. The data were filtered between 0.5 and 140 Hz and downsampled to 500 Hz.

Each task trial was epoched for encoding and maintenance period: (a) Encoding from −500ms to 1 s after the first sound item and the second sound items (i.e., resulting in two epochs per task trial); (b) Maintenance from 0 to 2.5 s after the memory cue; (c) Impulse sound period from −0.5 s to 1.5 s after onset of the impulse sound or the corresponding time point in trials with no impulse sounds. Epochs were rejected if the peak-to-peak amplitude during the epochs exceeded 1000 fT and 3000 fT/cm in any of the magnetometer and gradiometer channels, respectively. On average, we had 115 ± 15 epochs for encoding and 58 ± 7 epochs for maintenance for each ripple-velocity condition. One subject was excluded due to excessive motion noise, resulting in 19 subjects in total. Two additional subjects were excluded from the connectivity analysis because of an insufficient number of epochs to provide a sufficient signal to noise ratio for coherence analyses.

#### Source estimation

The geometry of each participant’s cortical surface was reconstructed from the 3D structural MRI data using FreeSurfer software (https://surfer.nmr.mgh.harvard.edu). The cortical surface was decimated to a grid of 10242 dipoles per hemisphere, corresponding to a spacing of approximately 5 mm between adjacent source locations in the cortex. The MEG forward solution was computed using a single-compartment boundary-element model (BEM) assuming the shape of the intracranial space. The watershed algorithm was used to generate the inner skull surface triangulations from the T1-weighted MRIs of each participant. The cortical current distribution was estimated using minimum-norm estimate (MNE) software (http://www.nmr.mgh.harvard.edu/martinos/userInfo/data/sofMNE.php) (Gramfort et al., 2014) and assuming the orientation of the source to be fixed perpendicular to the cortical mesh. The noise-covariance matrix used to calculate the inverse operator was estimated from data collected without a subject present. To reduce the bias of the MNEs toward superficial currents, we used depth weighting (Lin et al., 2006).

#### Inter-subject cortical surface registration for group analysis

Each participant’s inflated cortical surface was registered to an average cortical representation (fsaverage in FreeSurfer) by optimally aligning individual sulcal-gyral patterns (Fischl et al., 1999).

#### Region of interest (ROI) identification and analysis

We selected the ROIs based on the Freesurfer Desikan parcellation (Desikan et al., 2006), as guided by previous studies human auditory WM (Buchsbaum et al., 2005; Crottaz-Herbette et al., 2004; Huang et al., 2013; Kumar et al., 2016; Rodriguez-Jimenez et al., 2009; Uluç et al., 2018; Vallar et al., 1997). The combination of Heschl’s and superior temporal gyri was utilized to model auditory cortical WM processes (jointly labeled as STC). The other ROIs include the inferior frontal gyrus (IFG; pars opercularis, triangularis, and orbitalis), as well as the caudal middle frontal (CMF), lateral orbitofrontal (LOF), rostral middle frontal (RMF), precentral (PC), and supramarginal (SM) areas. An automatic routine was utilized to break each larger ROI into smaller, approximately equally sized sub-ROIs (Mamashli et al., 2019a). The purpose of this procedure was to increase the spatial specificity of our subsequent analyses of spatio-spectrotemporal WM activation patterns, as well as to deal with potential signal cancellations due to the sulcus geometry (Mamashli et al., 2019a). Furthermore, dividing each ROI into sub-ROI approach would allow us to account for the sensitivity of different parts of STC to different spectrotemporal properties (Schönwiesner and Zatorre, 2009) and testing our hypothesis of sound content-specific representation in the STC-ROI sub-regional phase-synchronization patterns.

#### Sub-ROI time series extraction

Epochs were extracted for all vertices within each sub-ROI using inverse operator. The time series were averaged across the vertices within every sub-ROI, with the waveform signs of sources aligned on the basis of surface-normal orientations to avoid phase cancellations. This results in a two-dimensional (2D) time-series matrix of epochs by time for each sub-ROI (Mamashli et al., 2018, 2019a).

#### Time-frequency decomposition

The 2D time series was convolved with a dictionary of complex Morlet wavelets (each spanning seven cycles), resulting in three-dimensional complex spectra epoch-time-frequency matrix: $S_{k}(t,f)\in R^{K\;\times \;T\times \;F}$, *K* is number of epochs, *T* is time points and *F* is frequency bins.

#### Functional connectivity computation

The coherence between each sub-ROI pair (*I_i_, J_j_*) in *ROI_I_* and *ROI*_*J*_ (*I* = 1, 2 and *J* = 1, …, 6), was computed for all frequencies between 3 Hz and 120 Hz across each 1/4^th^ of the total epochs. Functional connectivity was estimated for all intra-hemispheric connections with right STC and left STC (*I* = 1, 2): In summary, we had 6 STC-ROI connections in right hemisphere and 6 STC-ROI connections in left hemisphere. We used the imaginary part of the coherence (ImCoh) to minimize spurious connectivity due to field spread effect (Nolte et al., 2004). To maintain a constant signal-to-noise ratio across conditions, the number of epochs per condition per participant was fixed at the minimum number of accepted epochs that we had for each condition and participant. The connectivity analysis was not done for the impulse sound responses, as the impulse sound was present only in 50% of the epochs, which rendered the number of epochs too small to provide a sufficient signal to noise ratio of the coherence estimation.

#### Power computation

- MVPA analysis: Power spectrum (no temporal resolution) was estimated using multi-taper approach implemented for single epochs in MNE-Python software from 3 to 120 Hz for each sub-ROI. For each subject, the minimum number of epochs across conditions was considered to control for signal to noise ratio across conditions. Power spectrum was averaged into four groups for each condition. This allowed for four-fold cross validation in the subsequent multivariate pattern analysis (MVPA).
- Power variation during maintenance: Temporally resolved power values were averaged across 6-ripple velocity memorization during maintenance. Averaged power changes in the post stimulus interval (0 to 2 s) were estimated as relative change from the baseline interval (−400 to −200ms). Power values were averaged within each of the five studied frequency bands.

#### Machine learning analysis

MVPA have been successfully used to decode neural activity using MEG signal patterns (Haxby et al., 2014; King and Dehaene, 2014; Mohsenzadeh et al., 2018). We used MVPA to decode the six ripple sounds classes from MEG signal patterns during WM processing. Following our hypothesis, we used STC-ROI connectivity patterns and sub-regional power pattern within each ROI as our features.

- STC-ROI Connectivity Pattern: Connectivity pattern was extracted for both WM maintenance period and WM encoding period separately. In WM maintenance, we considered two time windows: early (0.5 to 1.25 s) and late (1.25 – 2 s) to allow us to investigate the stability of connectivity-based WM coding across the maintenance period. ImCoh values between each sub-ROI pair were averaged within each time window separately in each frequency band. In WM encoding ImCoh were averaged from 0-1 s. The frequency range used for each frequency bands were as follows: Theta: 3-7 Hz, Alpha: 8-12 Hz, Beta: 13-30 Hz, Low Gamma: 31-60 Hz and High Gamma: 61-120 Hz (Figure 2A). The STC-to-ROI connectivity matrix consisted of ***N*_*STC*_×*N*_*ROI*_**, (***N***_***STC***_: Number of sub-ROIs in STC, ***N***_***ROI***_: Number of sub-ROIs in ROI) sub-ROI pair connections, giving ***N*_*STC*_×*N*_*ROI*_** = ***T*** features for each frequency band (Figure 2B). The number of sub-ROIs in left and right STC were ***N***_***left STC***_ = **12** and ***N***_***right STC***_ = **13** respectively. The numbers of sub-ROIs in other ROIs are in Table S1. We used support vector machine (SVM) implemented in libsvm (Chang and Lin, 2011) and provided in the COSMOMVPA package (http://www.cosmomvpa.org/) (Oosterhof et al., 2016) in MATLAB. SVM is widely used in comparable neuroimaging studies because of its suitability for analyses with relatively small number of samples (< 10,000). Another advantage of linear SVM over other suitable classifiers, such as linear discriminant analysis, is the regularization parameter (known as C in the equation), which helps to adjust or penalize for the large number of features when estimating the cost function in the optimization process (Chang and Lin, 2011). SVM can be described as a hyperplane that separates the classes as best as possible. Specifically, we trained a SVM classifier with linear kernel and cost equal to one (***C*** = **1**). To increase the impact of the analysis to larger population, we merged the data from all subjects together and performed the classification across the subjects. In total, we had **306×*T*** dataset for training and tested on **102×*T*** dataset and did cross validation by permuting the whole data 100 times and replicating the classification process. Accuracy of the classifier as performance measures was averaged across 100 cross-validations. We also looked at the confusion matrix that was averaged across 100 cross-validation. This process was done for each STC-ROI connection and each frequency band. Therefore, we performed 60 (5 frequency bands X 12 connections) classifications procedure.
- ROI Power Pattern: We extracted sub-regional power pattern in WM encoding and WM maintenance separately. We used frequency specific power pattern within each frequency band (theta, alpha, beta, low gamma, and high gamma) in each ROI to decode the relevant ripple velocity class (out of six). The frequencies were selected logarithmically between 3 to 120 Hz with total 73 frequency bins. Therefore, there were ***N*×*M*** = ***H*** features (***N*** number of sub-ROI within each ROI and M frequency bins within frequency band, ***M*** = 5 in theta and alpha, ***M*** = 18 in beta, ***M*** = 20 in low gamma and ***M*** = 21 in high gamma). Power values within each sub-ROI were averaged across each 1/4^th^ of the epochs for each condition (Figure 5a) that allows four-fold cross validation (trained on **18×*H*** data and tested in **6×*H*** data, repeated 4 times) within each subject. The classifier was SVM with a “radial basis function” kernel as implemented in scikit-learn (Pedregosa et al., 2011) with default parameters; C = 1 and gamma is $\frac{1}{\left\{H\times \sigma^{2}(P)\right\}}$, in which **P** stands for power. The idea for this analysis was to (i) understand the effect of impulse sound in auditory cortex. Thus, we focused on the bilateral STC frequency specific power pattern and compared the decoding accuracies during the last 1.5 s of maintenance, across the trials with and without the impulse sound. (ii) Identify potential confounding effects in the MVPA connectivity analysis (Lowet et al., 2016) tested across all ROIs.
- Cortical Activation Temporal Decoding: We used the time course activation within each vertex in the whole cortex for decoding the represented ripple velocity during WM encoding and maintenance (Figure S6). We also compared the time-course decoding accuracies during the last 1.5 s of maintenance, across the trials with and without the impulse sound. We used the same time interval as the connectivity in both WM maintenance (0.5-2 s) and encoding (0-1 s) data. The data were band pass filtered from 0.5-12 Hz. During WM encoding, we had 500 samples and during maintenance 750 samples data both before and after impulse sound. We used principal component analysis (PCA) to reduce the number of temporal features from 750 and 500 time points to 100 principal components that explained 99% of the variance of the data. The input data to the classifier was ***N*_*trials*_×100** principal components for each subject. SVM classifier with radial basis function kernel was used with default value of C = 1, gamma = $\frac{1}{\left\{H\times \sigma^{2}(\mathrm{PC})\right\}}$, in which **PC** is the principal components, and 10-fold cross validations. We used equal number of trials across the conditions within each subject.

#### Similarity pattern between WM encoding and maintenance

MVPA STC-ROI Connectivity pattern: To test whether there were similarities in WM encoding and maintenance, we used a multivariate approach. We tested whether the connectivity pattern of the 6-ripple velocity memorized in “WM encoding” is able to predict the ripple velocity class in “WM maintenance.” To this end, we trained a linear SVM model with WM encoding connectivity pattern as features and tested with WM maintenance connectivity pattern with 4-fold cross validation and non-overlapping runs, as implemented across all connections and all frequency bands within each subject.

#### Behavioral relevance of functional connectivity during WM maintenance

In previous studies, direct correlations between MVPA decoding accuracy and behavioral WM performance have often been relatively scarce (Christophel et al., 2018; Kumar et al., 2016; Uluç et al., 2018), or they have emerged only in simpler tasks than that used in the present study (Bettencourt and Xu, 2016; Ester et al., 2013). These difficulties in finding direct correlations between MVPA and behavioral WM measures may stem from both cognitive (the additional contribution of recall/matching, decision making processes) and neuroimaging signal-processing (e.g., individual variable SNR) confounds. An inherent property of our between-subject connectivity-based SVM analysis, further, is that it outputs only group-level decoding accuracies, which limits correlation analyses to the variability of behavioral performance. Here, to examine the behavioral relevance of our connectivity-based measures, we therefore adapted a different strategy: The relationship between each subject’s proportion correct responses and functional connectivity patters was analyzed during the early (0.5 to 1.25 s) and late (1.25 – 2 s) maintenance time windows using a support vector regression (SVR) implemented in libsvm (Chang and Lin, 2011) and provided in the COSMOMVPA package (http://www.cosmomvpa.org/) in MATLAB.

Specifically, ImCoh values between each sub-ROI pair were averaged within each time window separately in the same frequency ranges that were utilized in the content-decoding analysis, including Theta (3-7 Hz), Alpha (8-12 Hz), Beta (13-30 Hz), Low Gamma (31-60 Hz), and High Gamma (61-120 Hz). Each subjects STC-to-ROI connectivity matrix consisted of *N*_*STC*_×*N*_*ROI*_ sub-ROI pair connections (*N*_*STC*_ is the number of sub-ROIs in STC, *N*_*ROI*_ refers to those in the other ROI; see Table S1) giving *N*_*STC*_×*N*_*ROI*_ = *T* features for each of the four subsamples and frequency band. The run-specific sub-ROI pair connectivity matrices were concatenated across subjects: The initial input data to our SVR, which used a linear kernel and cost equal to one (*C* = 1)), thus was (*N*_*runs*_×*N*_*subjects*_×*T* = 68×*T*. However, in each of our four cross-validation folds, the 51×*T* initial training set was subjected to a principal component analysis (PCA) to reduce its connectivity features to the number of principal components (PC) that explained 95% of the variance of the data. The resulting PCA coefficients were, subsequently, used to multiply the remaining test data, to yield a matching *N*_*PC*_ of features across the 17 subjects having a sufficient number of epochs for the connectivity analysis. The prediction accuracy was defined based on the root mean square error of the predicted versus the actual proportion correct values (RMSE).

### QUANTIFICATION AND STATISTICAL ANALYSIS

- MVPA Connectivity analysis: We used nonparametric permutation approach to test the significance of the accuracy values. First, we created 500 unique permutations of the true labels of the classifier. A null distribution of the accuracy was generated using the training data with randomized item-content labels. This null distribution helped us determine the classification accuracies that emerge by chance with 6-classes. The null distribution was generated for each connection and frequency bands separately. To correct for multiple comparisons across all connections and frequency bands (total 60), we used maximum statistics. We took the maximum value of the null distribution across all 60 tests which provided a final null-distribution. To assign a p value for each connection, the original accuracy value (found from a classifier with true labels) was compared with this null-distribution.
- MVPA Power analysis: Similar to MVPA connectivity we followed a permutation approach. We generated a null distribution of accuracies with a classifier with randomized labels. We replicated this process for each subject and averaged the values. This was done separately for each frequency band. Lastly, we took the maximum across 5 frequency bands to correct for multiple comparisons. In addition, to compare decoding accuracies from with and without the impulse sound, we used LME modeling, as implemented using the MATLAB functions fitlme. The best fitting model was selected in a stepwise fashion by using likelihood ratio tests (compare.m): The complexity of the model was increased starting from the simplest possible model, which contained only the intercept and the random effect of subject identity, toward the full factorial model (containing all possible main effects and interactions) until we reached a point where no significant improvement was achieved. In addition to the random effect of subject identity and intercept, the LME analysis considered the fixed effects of Impulse Sound (impulse sound versus no impulse sound), the MEG Frequency Band (represented by each band’s center frequency) that was transformed by base-2 logarithm before the analysis. For the practical implementation, the accuracy values were rescaled by subtracting 1/6. We corrected for multiple comparisons by applying Bonferroni correction across 13 t statistics.
- Power variation during maintenance: threshold-free clustering was applied with one-sample t test as the test statistics and 500 permutations across 19 subjects.
- Cortical activation temporal decoding: To assess statistical significances, we used cluster-based statistics with 1,000 permutations, initial alpha value of 0.05 and one-sample t test as test statistics comparing the decoding accuracy against the chance level of 1/6 in whole cortex across all subjects (n = 19). Cluster-based statistics is a nonparametric, permutation-based method (Maris and Oostenveld, 2007) that inherently corrects for multiple comparisons. The cluster statistics was used to compare the decoding accuracy against the chance level during WM encoding, WM maintenance, and impulse sound response.
- Behavioral relevance of functional connectivity during WM maintenance: The RMSE values averaged across the four folds were compared to a null distribution, which was accumulated by repeating the same SVR procedure in 1000 permutations with training-set labels (i.e., proportion correct values) being randomly shuffled within each subsample. To manage the multiple comparisons problem, from each permutation, we selected the minimum RMSE across all sub-ROI frequency connectivity pairs, across the early and late time windows, to be entered into the null distribution. The alpha was set to p < 0.05 (two tails): the sub-ROI frequency connectivity patterns whose RMSE value was smaller than in 97.5% of those in the combined null distribution during both the early and late time window were deemed as statistically significant predictors of the behavioral performance.

## Supplementary Material

1

## Figures and Tables

**Table:** KEY RESOURCES TABLE

REAGENT or RESOURCE	SOURCE	IDENTIFIER
Deposited data		
MEG connectivity data	This study	https://dataverse.harvard.edu/citation?persistentId=doi:10.7910/DVN/I307DS
Software and algorithms		
MNE	Gramfort et al., 2014	https://www.nmr.mgh.harvard.edu/martinos/userInfo/data/sofMNE.php
FreeSurfer	Dale et al., 1999; Fischl et al., 1999	https://surfer.nmr.mgh.harvard.edu/
libsvm	Chang and Lin, 2011	https://www.csie.ntu.edu.tw/~cjlin/libsvm/
COSMOMVPA	Oosterhof et al., 2016	http://www.cosmomvpa.org/
Custom code	This study	https://zenodo.org/record/5112421

## Highlights

- How neurons in human brain store transient working memories (WMs) is debated
- We hypothesize that interregional phase synchronization supports WM maintenance
- We decode auditory memories from magnetoencephalograms to test this hypothesis
- Synchronization patterns across sensory and higher areas reveal content held in WM
