# Supplementary material for: Synchronization patterns reveal neuronal coding of working memory content
Source: Cell Rep. Author manuscript; Available in PMC 2021 Sep 9. (PMC8428113; doi:10.1016/j.celrep.2021.109566)
Supplement: 1 [file NIHMS1735642-supplement-1.pdf]

Cell Reports, Volume *36*

Supplemental information

Synchronization patterns reveal neuronal coding of working memory content

Fahimeh Mamashli, Sheraz Khan, Matti Hämäläinen, Mainak Jas, Tommi Raij, Steven M. Stufflebeam, Aapo Nummenmaa, and Jyrki Ahveninen

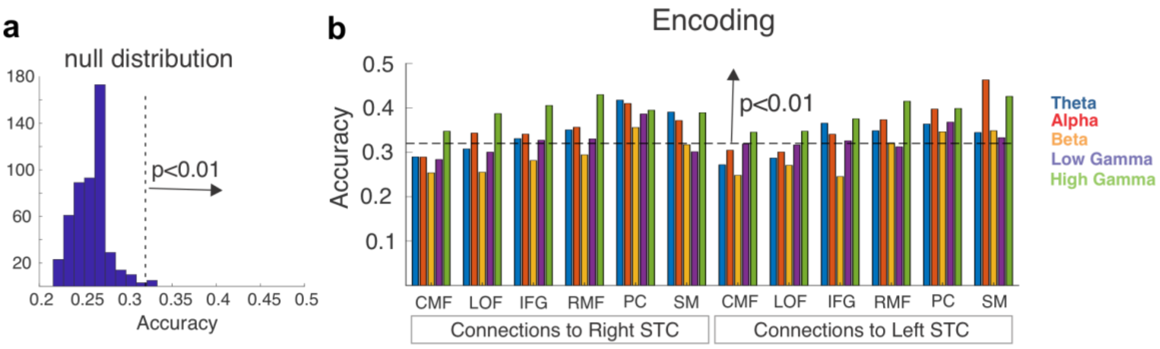

Figure S1: *Decoding accuracy in the connectivity-based MVPA at the five studied frequency bands during WM encoding*. Related to Figure 3. (a) The null distribution of accuracy that was created using a classifier with randomized labels. The threshold of significance is marked. (b) Accuracy values of each connection and each frequency band. Those above the threshold (dashed line) are significant. For details of the analysis procedure, see STAR Methods.

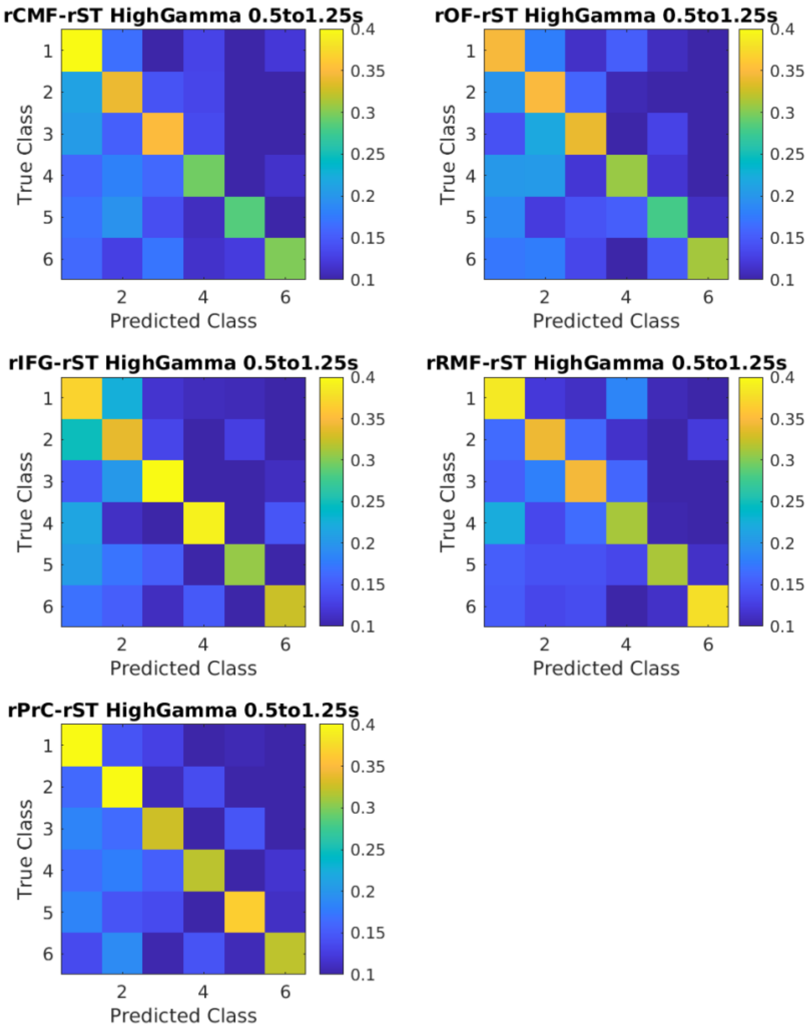

Figure S2: *Normalized confusion matrix for connections in high gamma band and right hemisphere for the early time interval of maintenance period*. Related to Figures 2 and 3.

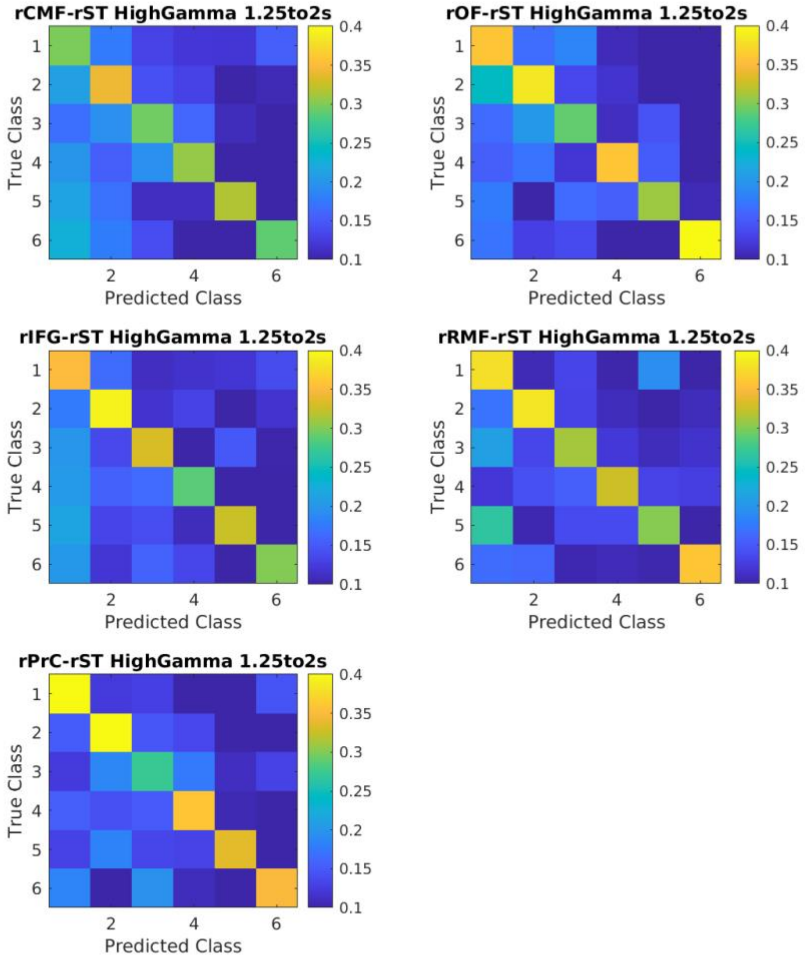

Figure S3: *Normalized confusion matrix for connections in high gamma band and right hemisphere for the later time interval of maintenance period*. Related to Figures 2 and 3.

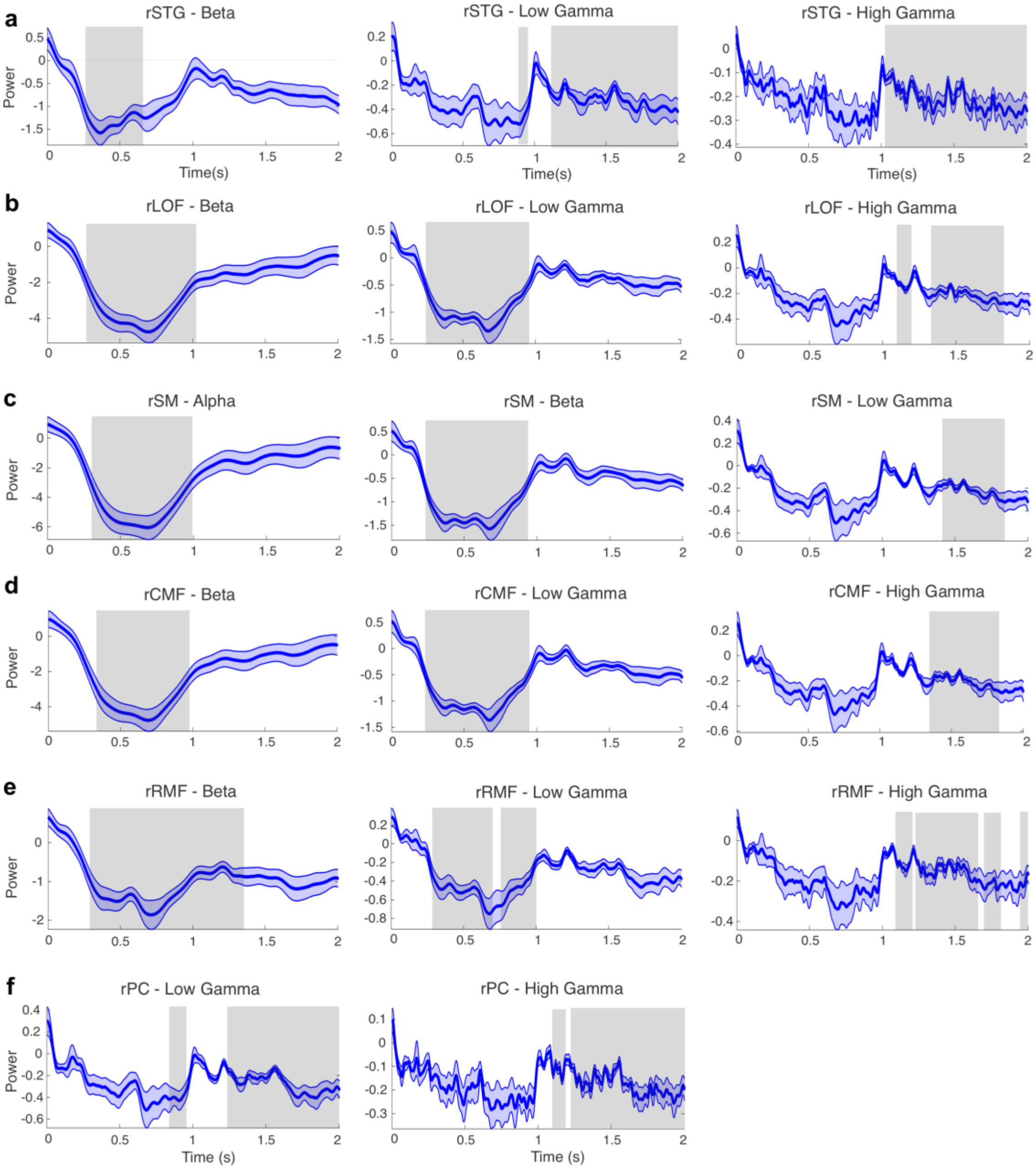

Figure S4: *Power variation in time during maintenance in right hemisphere*. Related to Figure 5. Mean power across all subjects in (a) rSTG, (b) rLOF, (c) rSM, (d) rCMF, (e) rRMF and (f) rPC. Power values were averaged across the six memorized sound. Threshold free clustering were used to find significant change from baseline (-400ms to -200ms prior to memorization period). The shaded gray area is the significant period and shaded blue area around the mean corresponds to one-standard error of mean. Title of each column shows the frequency band. For details of the analysis procedure, see STAR Methods.

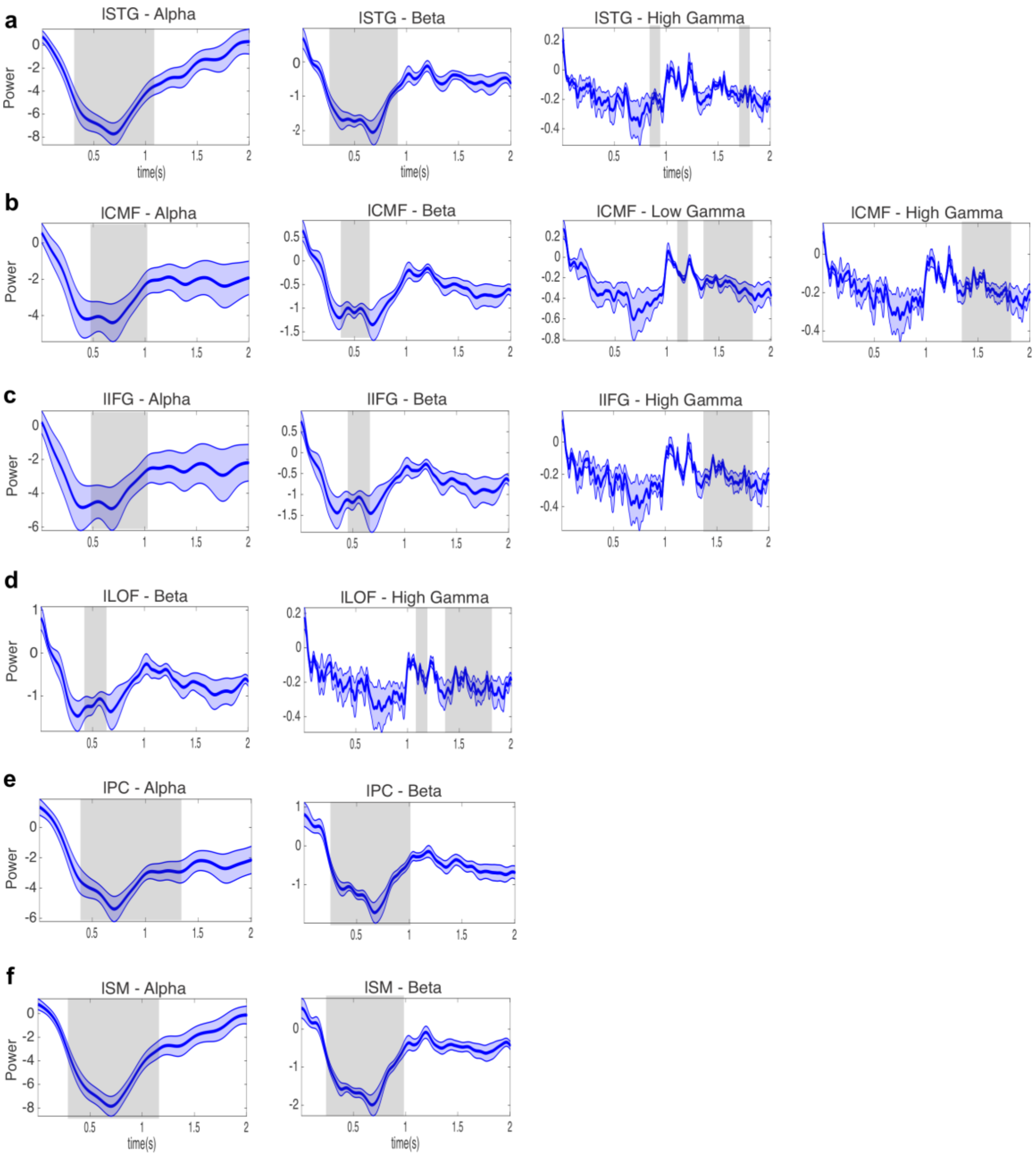

Figure S5: *Power variation in time during maintenance in left hemisphere*. Related to Figure 5. Mean power across all subjects in (a) lSTG, (b) lCMF, (c) lIFG, (d) lLOF, (e) lPC and (f) lSM. Power values were averaged across the six memorized sound. Threshold free clustering were used to find significant change from baseline (-400ms to -200ms prior to memorization period). The shaded gray area is the significant period and shaded blue area around the mean corresponds to one standard error of mean. Title of each column shows the frequency band. For details of the analysis procedure, see STAR Methods.

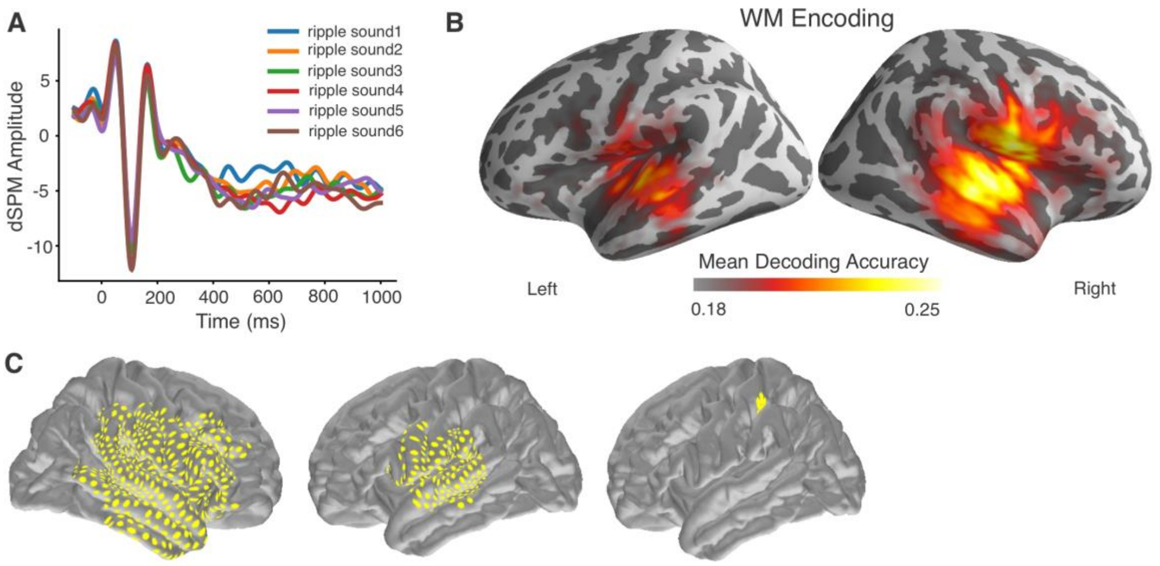

Figure S6: *Temporal pattern decoding.* Related to Figure 3. We tested whether the auditory WM content can be predicted using the local activity within each vertex. During the WM encoding, the six ripple-sound classes could be predicted at an accuracy that was significantly above the chance level. However, no significant effects were found during the maintenance period neither before impulse sound (0.5-2 second) nor after impulse sound (2.5-4 second). (A) Group-averaged evoked responses to the six ripple sound classes in the STC sub-ROI closest to the primary auditory cortex (medial Heschl's gyrus, i.e., the sub-ROI 1 in Figure 5A). (B) Mean decoding accuracy during WM encoding on the cortex (C) Significant cluster masks in WM encoding, which were found using, cluster statistics in the whole cortex comparing the decoding accuracy with chance level (1/6). For details of the analysis procedure, see STAR Methods.

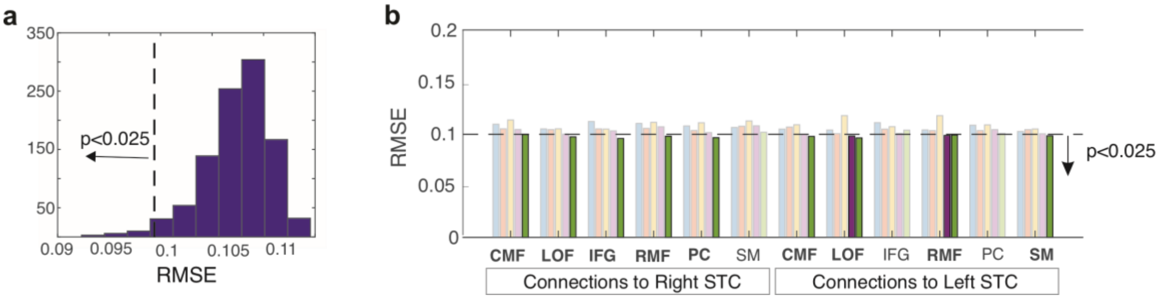

Figure S7: *Behavioral relevance of functional connectivity during WM maintenance*. Related to Figure 3. Prediction errors in the connectivity based SVR of behavioral performance are shown at the five studied frequency bands. (a) The null distribution of RMSE that was created using a SVR with randomized behavioral performance labels. The threshold of significance is marked. The details of statistical inference are described in STAR Methods. (b) RMSE of each connection and each frequency band. The solidly colored connections, where the RMSE is below the critical value marked by the horizontal dashed line, are statistically significant. Based on our SVR analyses and permutation testing, each subject's behavioral WM performance can, thus, be predicted based on their connectivity patterns during maintenance. Statistically significant and temporally consistent effects occurred mostly the at the high gamma range and to a lesser degree at the low gamma range, roughly mimicking the patterns observed in our analyses of content decoding. In the right hemisphere, statistically significant connectivity patterns that predicted the behavioral accuracy at the high gamma range STC vs. CMF, LOF, IFG, RMF, PC, and SM. The corresponding high-gamma connections in the left hemisphere included STC vs. LOF, IFG, RMF, and SM. At the low gamma range, significantly predictive connection patterns included those between the right STC vs. SM, as well as those between the left STC vs. IFG and RMF. Taken together, these SVR results demonstrate the behavioral relevance of frequency-specific synchronization patterns, which in our main analysis predicted the WM content at high accuracy. Significant effects concentrated at gamma range.

Table S1: The number of sub-ROIs within each ROI. Related to Figures 2-5 and STAR Methods.

ROI	Sub-ROI number
**rCMF**	5
**rOF**	7
**rIFG**	10
**rRMF**	13
**rPC**	16
**rSTC**	12
**lLOF**	7
**lIFG**	9
**lCMF**	6
**lRMF**	12
**lPC**	16
**lSTC**	13
**lSM**	10
**rSM**	9
**lLOC**	11
**rLOC**	10

Table S2: *Stepwise model selection results in STC for the analysis of impulse sound effects*. Related to Figure 3.

(a) The LME models in the order of complexity, as defined using Wilkinson notation: The simplest model (Model 1) considered the intercept and the random effect of identity. The next to simplest model (Model 2) considered also the fixed effect of Impulse sound. The subsequent model (Model 3) considered also the fixed effect of MEG Frequency Band. The most complex, i.e., the full model (Model 4), included also the interaction of Impulse Sound and MEG Frequency Band.

(b) The results of the model comparisons using the likelihood ratio test, based on the stepwise comparisons (i.e., 1 vs. 2, 2 vs. 3, 3 vs. 4). The best fit is achieved by Model 3, as no further improvement is achieved by the inclusion of the interaction term. Based on this best-fitting LME model, the content of auditory WM could be decoded significantly more accurately from STC from trials that included the impulse sound vs. those not including the impulse sound (t_{187}=4.0, $p_{Bonferroni}$=0.001). The best-fitting model, further, suggested that the decoding accuracy generally increased as a function of the increasing MEG frequency band (t_{187}=3.1, $p_{Bonferroni}$ =0.03). These results suggest that the impulse sound improves the decoding accuracy of WM content in STC areas that encompass the human auditory cortex. Further details of the analysis procedure are presented in STAR Methods.

a) Model definitions

Model 1: Decoding Accuracy ~ 1 + (1|Subject)
Model 2: Decoding Accuracy ~ 1 + Impulse + (1|Subject)
Model 3: Decoding Accuracy ~ 1 + Impulse+MEG *f* Band + (1|Subject)
Model 4: Decoding Accuracy ~ 1 + Impulse+MEG *f* Band + Impulse : MEG *f* Band + (1|Subject)

b) Likelihood-ratio test results

Model	DF	AIC	BIC	LogLik	LRStat	deltaDF	*p*
1	3	-402.5	-392.8	204.3			
2	4	-414.8	-401.8	211.4	14.3	1	0.0002
2	4	-414.8	-401.8	211.4			
3	5	-422	-405.8	216	9.2	1	0.0024
3	5	-422	-405.8	216			
4	6	-420.4	-400.9	216.2	0.3	1	0.5562

Abbreviations: DF, degrees of freedom; AIC, Aikeke Information Criterion; BIC, Bayesian Information Criterion; LogLik, Log likelihood; LRStat, likelihood ratio statistic; deltaDF, difference in DFs.
